# Autophagy: When to strike?

**DOI:** 10.33696/cancerimmunol.2.008

**Published:** 2020

**Authors:** Shadi Zahedi, Jean M. Mulcahy Levy

**Affiliations:** 1 Department of of Pediatrics, University of Colorado School of Medicine, Aurora, CO 80045, USA; 2 Morgan Adams Foundation Pediatric Brain Tumor Research Program, Children’s Hospital Colorado, Aurora, USA; 3 Department of Pharmacology, University of Colorado School of Medicine, Aurora, CO 80045, USA

Autophagy was originally viewed as a widely conserved multistep lysosomal degradation pathway in eukaryotes. It includes the formation of autophagosomes, double-membrane structures engulfing cytoplasm with damaged organelles during the degradation process. Under normal conditions, autophagy is a mechanism of cell survival, however, the role of autophagy in human disease remains complicated.

In cancer, autophagy seems to have a dual role in tumor cell survival and death. During early stages of tumorigenesis, autophagy can limit tumor growth, however, in advanced cancers it may facilitate tumor progression as a protective mechanism against various stress conditions [1]. Given that tumors are frequently exposed to environmental stresses such as nutrient deprivation, low PH and hypoxic conditions, inhibiting autophagy appears to be a promising target for therapy. In fact, we and others have shown that targeting this pathway in combination with existing therapies can improve therapeutic outcome in some cancers [2–6].

In addition, there are somatic mutations that would predispose sensitivity to autophagy inhibition in certain tumor types. We have previously shown that BRAF^V600E^ makes pediatric central nervous system (CNS) tumor cells sensitive to autophagy inhibition as they demonstrate high rates of autophagy compared to wild-type cells [2]. We also have demonstrated *in vitro, ex vivo,* and in patients that autophagy inhibition overcomes multiple molecularly distinct resistance mechanisms to BRAF inhibition in BRAF mutant CNS tumors. Particularly, there was a synergistic effect between BRAFi and autophagy inhibition [4]. Other groups have also shown the importance of autophagy in RAS mutant cancers as a key resistance mechanism to MEK or ERK inhibition. Combined autophagy inhibition in addition to ERKi and MEKi resulted in potent cytotoxicity in those models [5,6]. Current research efforts have mostly focused on utilizing chloroquine (CQ) or its derivatives such as hydroxychloroquine (HCQ) to inhibit late stage autophagy. However, lack of specificity, dose limiting cytotoxicity in combination with cytotoxic chemotherapy and inconsistency in autophagy inhibition across tumor types continues to be a challenge for the clinical use of these drugs [1]. Further studies have demonstrated differential effects of early versus late stage autophagy inhibition on tumor cell killing [7].

Together, these studies demonstrate how it is more essential to determine if inhibiting earlier phases of autophagy (involved in autophagosome formation) or later phases (involved in autophagosome cargo digestion) would yield better therapeutic outcomes. In our research studies, we aim to determine the optimal point to target and disrupt autophagy in BRAF^V600E^ brain tumor cells in order to improve patient outcomes. Our most recent data were able to demonstrate the effectiveness of early stage autophagy inhibition against ULK1 and VPS34, two early autophagy regulators, using SBI- 0206965 and VPS34-IN1 respectively [3]. Both genetic and pharmacologic inhibition of early stage autophagy, particularly in the presence of BRAFi, reduced tumor cell growth and enhanced tumor cell death in BRAF mutant CNS tumor cells irrespective of their RAFi sensitivity. Interestingly, we observed increased treatment efficacy using early stage autophagy inhibitors in cells under stress (nutrient deprivation) which mirrors the tumor microenvironment. Considering that others have shown a synergistic effect between ULKi inhibition and mTOR inhibition [8,9], additional studies will be important to determine if we could increase treatment efficacy using mTOR inhibitors in combination with these early stage autophagy inhibitors in CNS tumors. These data suggest early stage autophagy inhibition may be a viable target in autophagy dependent CNS tumors.

As more specific and optimized autophagy inhibitors are being developed, future studies will directly compare early and late stage autophagy inhibition to determine optimal targets in autophagy dependent BRAF mutant CNS tumors. Considering development of resistance to standard therapies remains a challenge even in combination targeted therapies, the need for developing the most effective combination therapies gains considerable importance. In combination with autophagy inhibition, studies to investigate targeting additional pathways such as those involved in other stress responses and even harnessing the immune response to improve treatment outcomes are important.

Initially, both cytotoxic innate and adaptive immune systems can control tumor development. Tumor-associated danger signals result in acute inflammatory responses leading to tumor cell recognition, cytokine secretion (specifically, interleukin-12 (IL-12) and interferon-γ (IFN-γ), and tumor cell killing by natural killer (NK) cells, dendritic cells (DCs), and macrophages. After migrating to nearby lymph nodes, Mature DCs present tumor antigens and activate CD4^+^ and CD8^+^ T cells which will then migrate to tumor site and facilitate tumor cell killing [10].

Some tumor cells may manage to evade immune system attacks through developing various mechanisms and replicate leading to clinically detectable tumors [11]. In addition to the contribution of hypoxic and immunosuppressive microenvironment, cancer cells may down-regulate tumor associated antigens (TAAs) and major histocompatibility complex (MHC) class I expression leading to the acquaintance of low immunogenicity [12]. Additionally, tumor cells may develop resistance by suppressing CD4^+^ and CD8^+^ T cells via immunosuppressive cytokines (such as IL-10), factors regulating lymphocyte chemotaxis or immune check points such as programmed cell death protein 1 (PD1) facilitating the differentiation of immunosuppressive regulatory T cells [13].

It has been reported that autophagy can regulate immune system components, in particular NK cells, DCs, and T and B lymphocytes. By influencing their survival, activation, proliferation, differentiation, and homeostasis, autophagy can affect innate and adaptive immune responses. For example, initiation of tumor growth has been associated with decreased autophagy and infiltration of regulatory T cells that suppress the immune system [14]. It can also impact the release of cytokines and antibodies. Cytokines can also stimulate the early stages of autophagy but block autophagy flux (or the completion of the cycle) which in turn aggravates ER stress and increases lysosomal cell death [15]. It is important to note that a number of cytokines, immunoglobulins, and immune-related cells in turn affect the function of autophagy. For instance, transforming growth factor (TGF)-β, IFN-γ, IL-1, IL-2, and IL-12 are considered as autophagy inducers and IL-IL-10, and IL-13 can act as autophagy inhibitors [16].

The exact role or interaction between autophagy and the body’s immune response to tumors remains in debate. On one side, it’s possible that effective autophagy is needed to stimulate tumor recognition by the immune system [17,18]. It has also been shown that autophagy supports antigen presentation and a potential improved immune response [19]. Inhibition of autophagy could, in theory, blunt these responses. In contrast, it has been shown that autophagy inhibition during immunotherapy can enhance sustained tumor regression [20]. Targeted autophagy inhibition in T-cells can enhance an antitumor immune response by increasing the shift to effector memory cells and increasing production of interferon-γ [21]. Studies utilizing both early and late stage autophagy inhibitors have demonstrated immune reactivation against tumors. For instance, a recent report showed that lysosomes limited anticancer efficacy of CD8^+^ T cells in melanoma. Also, in melanoma, upregulation of autophagy by hypoxia resulted in diminished cell death induced by immune effectors. Treatment with HCQ enhanced tumor cell killing under this hypoxic condition [22]. Studies have shown that beclin1, a key component of early stage autophagy, results in an increase in T cell infiltration into the tumor microenvironment [23]. Finally, there are studies that find an equivalent T-cell response with and without autophagy inhibition [24].

Even though immunotherapeutic strategies aimed at boosting anti-tumor immunity are promising, immune tolerance remains a major challenge in cancer immunotherapy. As immunologic tolerance molecules such as IDO, CTLA-4, and PD-1 can regulate immune tolerance through autophagy pathways, it is key to understand the relationship between autophagy and tumor immune tolerance to design the most effective treatment strategy [15]. For instance, PD-1, a T-cell inhibitory checkpoint molecule, interacts with PDL-1 on the surface of the tumor cells suppressing an anti-tumor response. Recent studies have shown that blocking PD-1/PDL-1 axis via anti-PD-1 and anti-PDL-1 antibodies triggers autophagy in tumor cells and the addition of autophagy inhibitors can serve as an attractive combination immunotherapy approach [25]. Other studies have demonstrated anti-PDL-1 as a potential biomarker for response to mTOR or autophagy inhibitors in selected cancers [25].

Although, emerging evidence from cancer immunotherapy clinical trials has highlighted the crucial role of T cells in tumor elimination, most encouraging results have been in the context of hematological cancers and more recently in melanoma. Improving responses in CNS tumors continues to be complex with additional issues such as how to traffic the appropriate immune cells from the periphery into the brain [26]. And once the correct cells are in the CNS, how do we make them work better? There is a clear, although complex, connection between autophagy and the tumor immune response. We have clearly shown that both early and late stage autophagy inhibition are effective in autophagy dependent CNS tumors, such as those with BRAF mutations [2,3]. But can these responses be improved with a better understanding of the link between these pathways and the immune system? Early studies in melanoma have already investigated triple therapy with BRAF, MEK and PD-1 blockade and shown improved tumor control [27]. Is it possible to further these responses with autophagy manipulation? Future studies are ongoing to answer these questions and it will be important to include the analysis of anti-tumor immune responses in ongoing and future clinical trials where we are manipulating autophagy.

## References

[1] Levy JM, Thorburn A. Autophagy in cancer: moving from understanding mechanism to improving therapy responses in patients. Cell Death & Differentiation. 2019 12 13:1–5. 10.1038/s41418-019-0474-7 PMC7206017 31836831

[2] Levy JM, Thompson JC, Griesinger AM, Amani V, Donson AM, Birks DK, Morgan MJ, Mirsky DM, Handler MH, Foreman NK, Thorburn A. Autophagy inhibition improves chemosensitivity in BRAFV600E brain tumors. Cancer Discovery. 2014 7 1;4(7):773–80. 24823863 10.1158/2159-8290.CD-14-0049 PMC4090283

[3] Zahedi S, Fitzwalter BE, Morin A, Grob S, Desmarais M, Nellan A, Green AL, Vibhakar R, Hankinson TC, Foreman NK, Levy JM. Effect of early-stage autophagy inhibition in BRAF V600E autophagy-dependent brain tumor cells. Cell Death & Disease. 2019 9 12;10(9):1–5. 10.1038/s41419-019-1880-y PMC6742667 31515514

[4] Levy JM, Zahedi S, Griesinger AM, Morin A, Davies KD, Aisner DL, Kleinschmidt-DeMasters BK, Fitzwalter BE, Goodall ML, Thorburn J, Amani V. Autophagy inhibition overcomes multiple mechanisms of resistance to BRAF inhibition in brain tumors. Elife. 2017 1 17;6:e19671. 10.7554/eLife.19671 PMC5241115 28094001

[5] Wang Y, Gallant RC, Ni H. Extracellular matrix proteins in the regulation of thrombus formation. Current opinion in hematology. 2016 5 1;23(3):280–7. 26871252 10.1097/MOH.0000000000000237

[6] Kinsey CG, Camolotto SA, Boespflug AM, Guillen KP, Foth M, Truong A, Schuman SS, Shea JE, Seipp MT, Yap JT, Burrell LD. Protective autophagy elicited by RAF -- MEK “-ERK inhibition suggests a treatment strategy for RAS-driven cancers. Nature Medicine. 2019 4;25(4):620–7. 10.1038/s41591-019-0367-9 PMC6452642 30833748

[7] Goodall ML, Fitzwalter BE, Zahedi S, Wu M, Rodriguez D, Mulcahy-Levy JM, Green DR, Morgan M, Cramer SD, Thorburn A. The autophagy machinery controls cell death switching between apoptosis and necroptosis. Developmental Cell. 2016 5 23;37(4):337–49. 27219062 10.1016/j.devcel.2016.04.018 PMC4886731

[8] Egan DF, Chun MG, Vamos M, Zou H, Rong J, Miller CJ, Lou HJ, Raveendra-Panickar D, Yang CC, Sheffler DJ, Teriete P. Small molecule inhibition of the autophagy kinase ULK1 and identification of ULK1 substrates. Molecular Cell. 2015 7 16;59(2):285–97. 26118643 10.1016/j.molcel.2015.05.031 PMC4530630

[9] Martin KR, Celano SL, Solitro AR, Gunaydin H, Scott M, O’Hagan RC, Shumway SD, Fuller P, MacKeigan JP. A potent and selective ULK1 inhibitor suppresses autophagy and sensitizes cancer cells to nutrient stress. Iscience. 2018 10 26;8:74–84. 30292171 10.1016/j.isci.2018.09.012 PMC6172447

[10] Wang M, Zhang C, Song Y, Wang Z, Wang Y, Luo F, Xu Y, Zhao Y, Wu Z, Xu Y. Mechanism of immune evasion in breast cancer. OncoTargets and Therapy. 2017;10:1561. 28352189 10.2147/OTT.S126424 PMC5359138

[11] Gonzalez H, Hagerling C, Werb Z. Roles of the immune system in cancer: from tumor initiation to metastatic progression. Genes & Development. 2018 10 1;32(19–20):1267–84. 30275043 10.1101/gad.314617.118 PMC6169832

[12] Sethumadhavan S, Silva M, Philbrook P, Nguyen T, Hatfield SM, Ohta A, Sitkovsky MV. Hypoxia and hypoxia-inducible factor (HIF) downregulate antigen-presenting MHC class I molecules limiting tumor cell recognition by T cells. PLoS One. 2017;12(11). 10.1371/journal.pone.0187314 PMC5695785 29155844

[13] Rabinovich GA, Gabrilovich D, Sotomayor EM. Immunosuppressive strategies that are mediated by tumor cells. Annual Review of Immunology. 2007 4 23;5:267–96. 10.1146/annurev.immunol.25.022106.141609 PMC2895922 17134371

[14] Rao S, Tortola L, Perlot T, Wirnsberger G, Novatchkova M, Nitsch R, Sykacek P, Frank L, Schramek D, Komnenovic V, Sigl V. A dual role for autophagy in a murine model of lung cancer. Nature Communications. 2014 1 20;5(1):1–5. 10.1038/ncomms4056 24445999

[15] Jiang GM, Tan Y, Wang H, Peng L, Chen HT, Meng XJ, Li LL, Liu Y, Li WF, Shan H. The relationship between autophagy and the immune system and its applications for tumor immunotherapy. Molecular Cancer. 2019 12;18(1):17. 30678689 10.1186/s12943-019-0944-z PMC6345046

[16] Harris J. Autophagy and cytokines. Cytokine. 2011 11 1;56(2):140–4. 21889357 10.1016/j.cyto.2011.08.022

[17] Ko A, Kanehisa A, Martins I, Senovilla L, Chargari C, Dugue D, Marino G, Kepp O, Michaud M, Perfettini JL, Kroemer G. Autophagy inhibition radiosensitizes in vitro, yet reduces radioresponses in vivo due to deficient immunogenic signalling. Cell Death & Differentiation. 2014 1;21(1):92–9. 24037090 10.1038/cdd.2013.124 PMC3857616

[18] Michaud M, Martins I, Sukkurwala AQ, Adjemian S, Ma Y, Pellegatti P, Shen S, Kepp O, Scoazec M, Mignot G, Rello-Varona S. Autophagy-dependent anticancer immune responses induced by chemotherapeutic agents in mice. Science. 2011 12 16;334(6062):1573–7. 22174255 10.1126/science.1208347

[19] Li Y, Hahn T, Garrison K, Cui ZH, Thorburn A, Thorburn J, Hu HM, Akporiaye ET. The vitamin E analogue a-TEA stimulates tumor autophagy and enhances antigen cross-presentation. Cancer Research. 2012 7 15;72(14):3535–45. 22745370 10.1158/0008-5472.CAN-11-3103 PMC3576035

[20] Liang X, De Vera ME, Buchser WJ, de Vivar Chavez AR, Loughran P, Stolz DB, Basse P, Wang T, Van Houten B, Zeh HJ, Lotze MT. Inhibiting systemic autophagy during interleukin 2 immunotherapy promotes long-term tumor regression. Cancer Research. 2012 6 1;72(11):2791–801. 22472122 10.1158/0008-5472.CAN-12-0320 PMC3417121

[21] DeVorkin L, Pavey N, Carleton G, Comber A, Ho C, Lim J, McNamara E, Huang H, Kim P, Zacharias LG, Mizushima N. Autophagy regulation of metabolism is required for CD8+ T cell anti-tumor immunity. Cell Reports. 2019 4 9;27(2):502–13. 30970253 10.1016/j.celrep.2019.03.037

[22] Pan H, Chen L, Xu Y, Han W, Lou F, Fei W, Liu S, Jing Z, Sui X. Autophagy-associated immune responses and cancer immunotherapy. Oncotarget. 2016 4 19;7(16):21235. 26788909 10.18632/oncotarget.6908 PMC5008281

[23] Janji B, Berchem G, Chouaib S. Targeting autophagy in the tumor microenvironment: new challenges and opportunities for regulating tumor immunity. Frontiers in Immunology. 2018 4 25;9:887. 29922284 10.3389/fimmu.2018.00887 PMC5996896

[24] Starobinets H, Ye J, Broz M, Barry K, Goldsmith J, Marsh T, Rostker F, Krummel M, Debnath J. Antitumor adaptive immunity remains intact following inhibition of autophagy and antimalarial treatment. The Journal of Clinical Investigation. 2016 12 1;126(12):4417–29. 27775547 10.1172/JCI85705 PMC5127685

[25] Robainas M, Otano R, Bueno S, Ait-Oudhia S. Understanding the role of PD-L1/PD1 pathway blockade and autophagy in cancer therapy. OncoTargets and Therapy. 2017;10:1803. 28367063 10.2147/OTT.S132508 PMC5370068

[26] Ratnam NM, Gilbert MR, Giles AJ. Immunotherapy in CNS cancers: the role of immune cell trafficking. Neuro-oncology. 2019 1 1;21(1):37–46. 29771386 10.1093/neuonc/noy084 PMC6303437

[27] Ribas A, Lawrence D, Atkinson V, Agarwal S, Miller WH, Carlino MS, Fisher R, Long GV, Hodi FS, Tsoi J, Grasso CS. Combined BRAF and MEK inhibition with PD-1 blockade immunotherapy in BRAF-mutant melanoma. Nature Medicine. 2019 6;25(6):936–40. 10.1038/s41591-019-0476-5 PMC8562134 31171879

